# Designing Special Nonmetallic Superalkalis Based on a Cage-like Adamanzane Complexant

**DOI:** 10.3389/fchem.2022.853160

**Published:** 2022-03-14

**Authors:** Ya-Ling Ye, Kai-Yun Pan, Bi-Lian Ni, Wei-Ming Sun

**Affiliations:** ^1^ Fujian Key Laboratory of Drug Target Discovery and Structural and Functional Research, The School of Pharmacy, Fujian Medical University, Fuzhou, China; ^2^ School of Chemistry and Materials Science, University of Science and Technology of China, Hefei, China

**Keywords:** superalkali, adamanzane, superatom, nonlinear optics, reducing matters

## Abstract

In this study, to examine the possibility of using cage-like complexants to design nonmetallic superalkalis, a series of X@3^6^adz (X = H, B, C, N, O, F, and Si) complexes have been constructed and investigated by embedding nonmetallic atoms into the 3^6^adamanzane (3^6^adz) complexant. Although X atoms possess very high ionization energies, these resulting X@3^6^adz complexes possess low adiabatic ionization energies (AIEs) of 0.78–5.28 eV. In particular, the adiabatic ionization energies (AIEs) of X@3^6^adz (X = H, B, C, N, and Si) are even lower than the ionization energy (3.89 eV) of Cs atoms, and thus, can be classified as novel nonmetallic superalkalis. Moreover, due to the existence of diffuse excess electrons in B@3^6^adz, this complex not only possesses pretty low AIE of 2.16 eV but also exhibits a remarkably large first hyperpolarizability (*β*_0_) of 1.35 × 10^6^ au, indicating that it can also be considered as a new kind of nonlinear optical molecule. As a result, this study provides an effective approach to achieve new metal-free species with an excellent reducing capability by utilizing the cage-like organic complexants as building blocks.

## Introduction

Reducing agents with low ionization energies (IEs) play a crucial role in chemical synthesis. As is well-known, alkali metal atoms possess the lowest ionization energies (5.39–3.89 eV) (Lide, 2003) among all the elements in the periodic table. However, it is reported that a class of extraordinary compounds possesses even lower IEs than those of alkali metal atoms. Such species were termed “superalkalis” by Gutsev and Boldyrev (1982). Initially, superalkalis were designed by decorating an electronegative central atom with alkali-metal ligands, such as FLi_2_, OLi_3_, and NLi_4_ following the formula ML_*k*+1_ (L is an alkali-metal atom and M is an electronegative atom of valency *k*). In ML_*k*+1_, one more alkali metal atom will bring an extra valence electron for the electronic shell of M according to the octet rule. Consequently, such an ML_*k*+1_ complex has a great tendency to lose the extra valence electron and thus possess strong reducibility (Sun and Wu, 2019).

Owing to their excellent reducing ability, superalkalis can be used to synthesize unusual charge-transfer salts (Zintl and Morawietz, 1938; Jansen, 1976) with the counterpart possessing relatively low electron affinity and activate stable CO_2_ and N_2_ molecules (Park and Meloni, 2017; Zhao et al., 2017; Park and Meloni, 2018; Sun et al., 2019; Sikorska and Gaston, 2020) to produce high-value products (Zhang et al., 2021a; Zhang et al., 2021b). In particular, as a special subset of superatom (Reveles et al., 2009; Luo and Castleman, 2014), superalkalis can behave as alkali metal atoms and maintain their structural and electronic integrities when assembled into extended nanostructures (Reber et al., 2007). Hence, they offer an exciting prospect of serving as building blocks for nanomaterials with highly tunable properties (Jena and Sun 2018), such as supersalts (Giri et al., 2014), hydrogen storage materials (Merino et al., 2012), noble-gas-trapping agents (Pan et al., 2013), superbases (Srivastava and Misra, 2015), and nonlinear optical materials (Sun et al., 2014a; Sun et al., 2014b; Sun et al., 2016b; Sun et al., 2016c; Sun et al., 2018a).

In view of the great importance of superalkalis in chemistry, various superalkalis have been theoretically (Tong et al., 2009; Tong et al., 2011, Tong et al., 2012a,Tong et al., 2012b; Hou et al., 2013; Liu et al., 2014; Sun et al., 2013; Sun et al., 2016a; Giri et al., 2016; Zhao et al., 2017; Sun et al., 2018b; Sun et al., 2019; Park and Meloni, 2018; Sun and Wu, 2019; Tkachenko et al., 2019; Sikorska and Gaston, 2020) and experimentally (Lievens et al., 1999; Yokoyama et al., 2000, 2001; Hou and Wang, 2020) characterized in the past decades. To date, conventional mononuclear ML_*k*+1_ superalkalis have been expanded to dinuclear (Tong et al., 2009; Tong et al., 2011) and polynuclear (Tong et al., 2012a; Tong et al., 2012b; Liu et al., 2014) superalkalis, aromatic superalkalis (Sun et al., 2013), Zintl-ion-based superalkalis (Giri et al., 2016; Sun et al., 2018b), hyperalkalis (Sun et al., 2016a), alkali-metal complexes (Tkachenko et al., 2019), and so on. More importantly, some alkali-metal-free superalkalis (Hou et al., 2014; Liu et al., 2016), particularly nonmetallic superalkalis (Hou et al., 2013; Srivastava, 2019a; Srivastava, 2019b), have been proposed in recent years. For example, Hou et al. (2013) designed a class of M_2_H_2*n*+1_^+^ (M = F, O, N, C for *n* = 1, 2, 3, 4, respectively) superalkali cations by using hydrogen atoms as ligands. Following a similar rule, the other two series of nonmetallic superalkali cations, namely, F_*n*_H_*n*+1_^+^ (*n* = 1–10) and C_*x*_H_4*x*+1_^+^ (*x* = 1–5), have been proposed by Srivastava (2019a, 2019b). These achievements demonstrate that the potential of designing superalkalis of new type is limitless and thereby motivate us to create more diverse superalkali species by using different rules and ligands to further enrich the superalkali family.

More recently, Tkachenko et al. (2019) reported the record low ionization potentials (1.70–1.52 eV) of alkali metal complexes with crown ethers and cryptands and defined them as superalkali species. In fact, such alkali metal complexes were previously named as electrides, a special kind of ionic solids with trapped electrons serving as anions (Dye, 2009). Hence, this work first built a bridge between superalkalis and electrides. However, it is known that crown ethers and cryptands are prone to be cleaved at the C-O bonds (Redko et al., 2002). Fortunately, analogous complexants, such as adamanzane (adz) (Redko et al., 2002) and aza-cage (aza222) (Kim et al., 1999) with only C-N linkages and no amine hydrogens are considerably stable to synthesize the crystalline salts, including alkalides (Kim et al., 1999; Redko et al., 2002) and electrides (Redko et al., 2005) at room temperature. Hence, it is highly expected that such complexants could also be used as excellent building blocks to design and synthesize new superalkalis.

**FIGURE 1:**
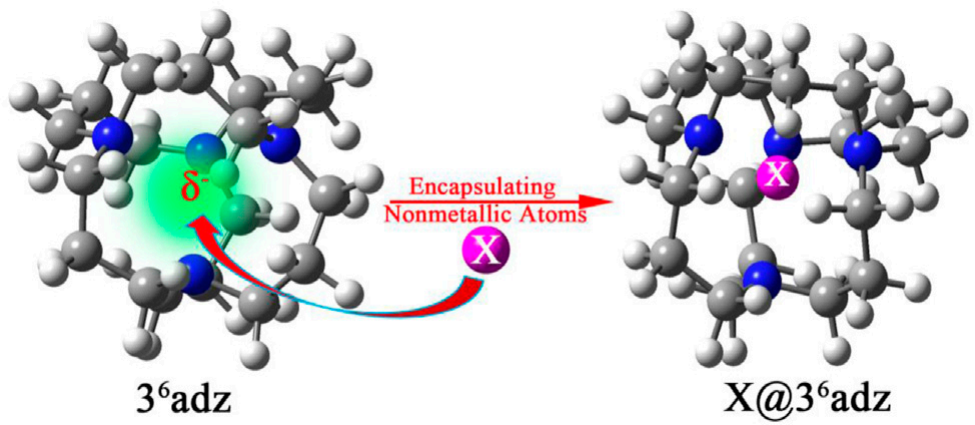
The schematic design strategy of X@3^6^adz (X = H, B, C, N, O, F, and Si) based on the cage-like 3^6^adz complexant.

To verify this hypothesis, the 3^6^adamanzane (3^6^adz) has been chosen as a representative to design a series of X@3^6^adz (X = H, B, C, N, O, F, and Si) by encapsulating nonmetallic atoms into the cavity of this cage-like complexant in this work (see Figure 1). The 3^6^adz complexant is composed of tricyclic tetra-amines with aliphatic chains (Springborg, 2003), which has been used to synthesize a stable alkalide [H@3^6^adz]^+^Na^−^ (Redko et al., 2002). In this complexant, all the lone pairs of 4 N atoms direct toward the center of the cage (see Supplementary Figure S1). Under the repulsion of the lone pairs of N atoms, the outmost valence electrons of X are destabilized to different degrees, leading to the obvious rise of HOMO level of X@3^6^adz as compared with the isolated 3^6^adz complexant. As a result, these proposed complexes exhibit extraordinarily low AIE values of 0.78–5.28 eV although X atoms possess very high ionization energies (IEs) of 8.15–17.42 eV (Lide, 2003). In particular, the B@3^6^adz complex also has the potential to serve as new nonlinear optical (NLO) molecule with a remarkably large first hyperppolarizability of 1.35 × 10^6^ au because the valence electron of boron atom is pushed out of cage to form diffuse excess electrons. We hope that this work will not only provide new nonmetallic members for the superatom family, but will also open the door to design strong reducing matters by embedding nonmetallic atoms into the various cage-like complexants.

## Computational Details

In this work, all the calculations were carried out by using the coulomb-attenuated hybrid exchange-correlation functional (CAM-B3LYP) (Tawada et al., 2004; Yanai et al., 2004), which has been reported to be capable of providing not only the molecular geometries close to the experimentally observed structures but also the (hyper)polarizabilities close to those of the coupled cluster calculations (Limacher et al., 2009). Hence, this method has been widely used to calculate the (hyper)polarizabilities of NLO molecules in the previous works (Sun et al., 2014a; Sun et al., 2014b, Sun et al., 2016c). Also, a method test has also been carried out by sampling B@3^6^adz (see Supplementary Table S1) to verify the reliability of this method in calculating the properties of such systems. From Supplementary Table S1, it is found that CAM-B3LYP gives approximately equal VIE and *β*_0_ to those obtained by several other functionals, which indicates that this method is reliable for these studied systems. Hence, all the optimized geometric structures of the studied species with real frequencies were obtained under the CAM-B3LYP/6-31+G(d) level. Based on the optimized structures, the single-point energies, nature population analysis (NPA) charges, and static electric properties were calculated at the CAM-B3LYP/6-311++G (d, p) level.

In this work, the vertical ionization energies (VIEs) of X@3^6^adz (X = H, B, C, N, O, F, and Si) were calculated as the energy difference between the optimized neutral complex and the cation in the geometry of the neutral complex, while their adiabatic ionization energies (AIEs) are defined as the energy difference between the neutral and cationic complex at their respective optimized structures. In addition, the TD-M06-2X calculations were performed to obtain the transition energies and oscillator strengths of the crucial excited states as well as the difference of the dipole moments between the ground state and crucial excited state of X@3^6^adz by using the 6-311++G (d, p) basis set. Herein, the dipole moments (*µ*_0_), polarizabilities (*α*_0_), and first hyperpolarizabilities (*β*_0_) are defined as follows,

$$\mu_{0}={\left(\mu_{x}^{2}+\mu_{y}^{2}+\mu_{z}^{2}\right)}^{1/2}$$ (1)

$$\alpha_{0}=\frac{1}{3}\left(\alpha_{\mathrm{xx}}+\alpha_{\mathrm{yy}}+\alpha_{\mathrm{zz}}\right)$$ (2)

$$\beta_{0}={\left(\beta_{x}^{2}+\beta_{y}^{2}+\beta_{z}^{2}\right)}^{1/2}$$ (3)

where $\beta_{i}=\frac{1}{3}\sum_{j}\left(\beta_{ijj}+\beta_{jji}+\beta_{jij}\right)$ , *i, j* = {*x, y, z*}.

All the above calculations were performed by using the GAUSSIAN 16 program package (Frisch et al., 2016). The dimensional plots of the molecular structures were generated with the GaussView program (Dennington et al., 2016).

## Results and Discussion

**FIGURE 2:**
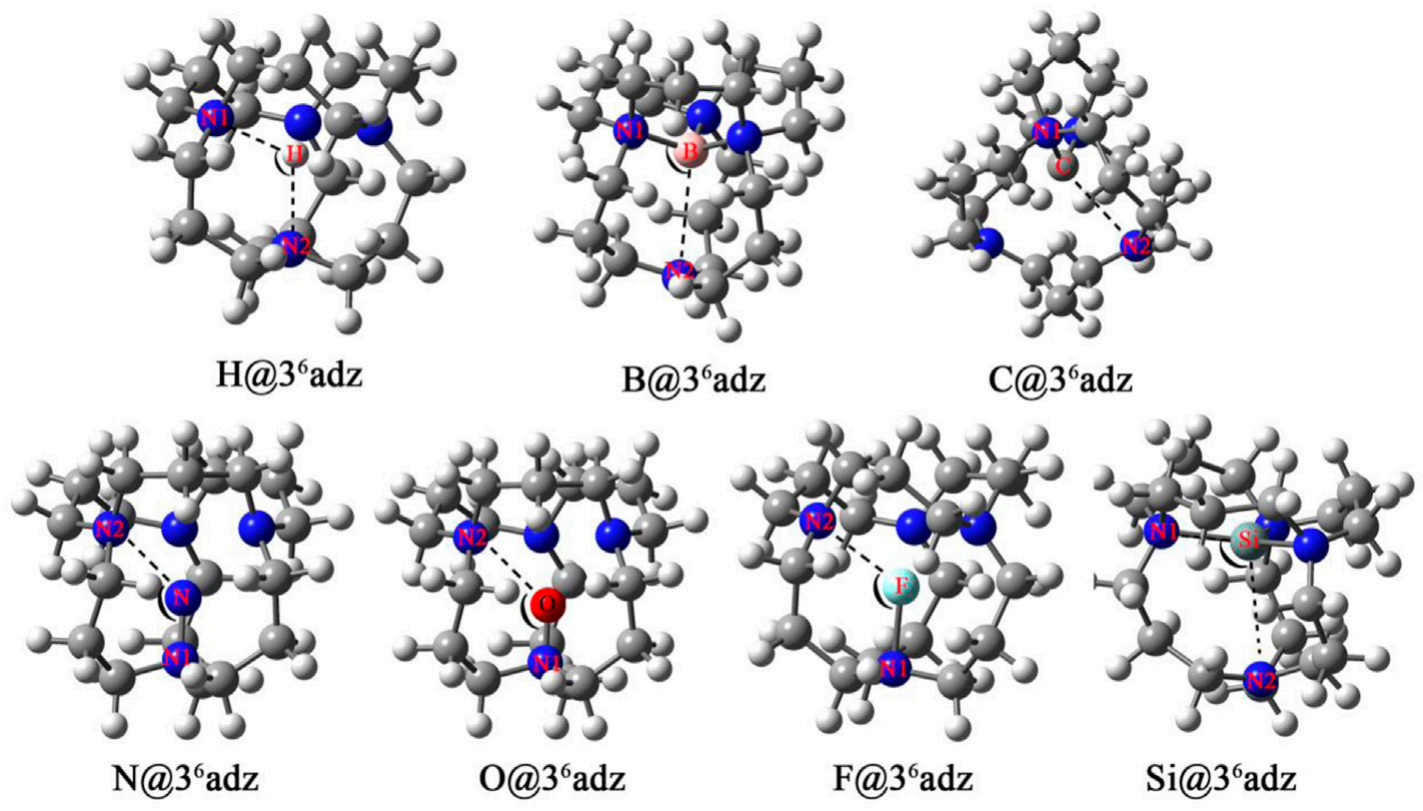
Optimized geometric structures of X@3^6^adz (X = H, B, C, N, O, F, and Si) compounds.

Initially, seven X@3^6^adz (X = H, B, C, N, O, F, and Si) compounds have been constructed by encapsulating one X atom into a 3^6^adz cage. After optimization, the geometric structures of X@3^6^adz are illustrated in Figure 2, while the corresponding cations are plotted in Supplementary Figure S2. Moreover, selected structural parameters of these resulting X@3^6^adz compounds are summarized in Table 1.

**TABLE 1:** Symmetry point group, the lowest vibrational frequencies *v*_1_ (in cm^−1^), the bond lengths of X-N1 and X-N2 bonds (*d*_X-N1_ and *d*_X-N2_, in Å), ∠N1-X-N2 angle (in deg) of X@3^6^adz (X = H, B, C, N, O, F, and Si) compounds.

Species	Symmetry	*v*_1_	*d*_X-N1_	*d*_X-N2_	∠N1-X-N2
H@3^6^adz	*S*_4_	69	2.11	2.11	113.5
B@3^6^adz	*C*_1_	91	1.66	3.02	105.9
C@3^6^adz	*C*_2_	102	1.52	2.97	108.4
N@3^6^adz	*C*_1_	48	1.41	2.58	120.3
O@3^6^adz	*C*_1_	72	1.34	2.59	125.1
F@3^6^adz	*C*_1_	61	1.87	2.41	123.7
Si@3^6^adz	*C*_1_	76	2.06	3.22	102.0

As shown in Figure 1, 3^6^adz is a cage-like complexant with *S*_4_ symmetry. From Figure 2, it is observed that the geometric integrity of 3^6^adz cage is well-preserved in these X@3^6^adz compounds. However, the geometric symmetries of these compounds are lowered to *C*_1_ and *C*_2_, except for H@3^6^adz, which maintains the *S*_4_ symmetry of 3^6^adz. To be specific, the encapsulated hydrogen atom located at the central position of 3^6^adz in H@3^6^adz, yields the newly formed N-H bonds of 2.11 Å and ∠N1-H-N2 of 113.5°. As for B@3^6^adz, the boron atom tends to bind with 3 N atoms of the complexant, forming 3 N-B bonds of 1.63 Å ∼ 1.66 Å, while the distance between the uncombined N and B atoms is as long as 3.02 Å. The C@3^6^adz complex possesses a *C*_2_-symmetric structure, where the introduced carbon atom prefers to bind with 2 N atoms of 3^6^adz by forming two N-C bonds of 1.52 Å. Differently, the more electronegative N, O, and F atoms are linked to only 1 N atom of the cage complexant *via* N-N, N-O, and N-F bonds of 1.41, 1.34, and 1.87 Å, respectively, generating the very similar structures of X@3^6^adz (X = N, O, and F). Similar to B@3^6^adz, the introduced silicon atom tends to bind with 3 N atoms of complexant *via* 3 N-Si bonds of 2.06–2.35 Å in Si@3^6^adz.

By turning to the cations of X@3^6^adz, it is found that only the optimized structure of [B@3^6^adz]^+^ cation almost coincides with the geometry of the corresponding neutral one (see Supplementary Figure S2). For instance, the critical geometric parameters of *d*_B-N1_, *d*_B-N2_, and ∠N1-B-N2 are hardly changed after one electron is lost from B@3^6^adz. However, for the rest of X@3^6^adz (X = H, C, N, O, F, and Si), quite different geometries of cationic and neutral complexes were found. For instance, the H^+^ is attached to 1 N atom of the complexant in the resulting [H@3^6^adz]^+^, while the doped N atom turns to combine with 2 N atoms of 3^6^adz in [N@3^6^adz]^+^ and Si atom almost moves to the center of the cage in [Si@3^6^adz]^+^. The geometry of C@3^6^adz is distorted from *C*_2_ symmetry to *C*_1_ with the changes of 0.29 Å for the C-N2 bond and 7.3° for ∠N1-C-N2. As for [F@3^6^adz]^+^, the N-F bond is shortened from 1.87 Å to 1.38 Å because the introduced F atom further loses 0.333*e* (see Supplementary Table S1) and thus tends to bind more tightly with the N atom of the complexant. Also, as shown in Table 2, the difference in the geometry can also be reflected by the difference of 0.29–3.06 eV between the vertical ionization energies (VIEs) and adiabatic ionization energies (AIEs) of these X@3^6^adz (X = H, C, N, O, F, and Si) species.

**TABLE 2:** Adiabatic ionization energies (AIEs, in eV), vertical ionization energies (VIEs, in eV), HOMO and LUMO energy levels (in eV), and the HOMO–LUMO gaps of 3^6^adz and X@3^6^adz (X = H, B, C, N, O, F, and Si) compounds.

Species	AIE	VIE	HOMO	LUMO	Gap(eV)
3^6^adz	6.56	6.80	−6.49	−0.38	6.12
H@3^6^adz	0.78	3.83	−3.49	0.36	3.86
B@3^6^adz	2.16	2.18	−1.81	−0.01	1.80
C@3^6^adz	2.72	3.01	−3.08	0.10	3.18
N@3^6^adz	3.15	5.72	−4.48	0.19	4.67
O@3^6^adz	5.28	5.86	−5.65	0.18	5.83
F@3^6^adz	4.92	6.38	−5.87	−0.13	5.73
Si@3^6^adz	1.79	2.73	−2.61	0.26	2.87

More interestingly, as shown in Table 2, extraordinarily low AIE values of 0.78–5.28 eV were found for all the studied X@3^6^adz (X = H, B, C, N, O, F, and Si) complexes, although X atoms possess very high ionization energies (IEs) of 8.15–17.42 eV (Lide, 2003). Such low AIE values of X@3^6^adz are not only lower than that of 6.56 eV for the 3^6^adz complexant but also significantly lower than that of 5.39 eV (Lide, 2003) for lithium atom. In particular, the AIE values of H@3^6^adz (0.78 eV), B@3^6^adz (2.16 eV), C@3^6^adz (2.72 eV), N@3^6^adz (3.15 eV), and Si@3^6^adz (1.79 eV) are even lower than the IE of 3.89 eV (Lide, 2003) for Cs atoms. Hence, these compounds should be classified as novel nonmetallic superalkalis.

**FIGURE 3:**
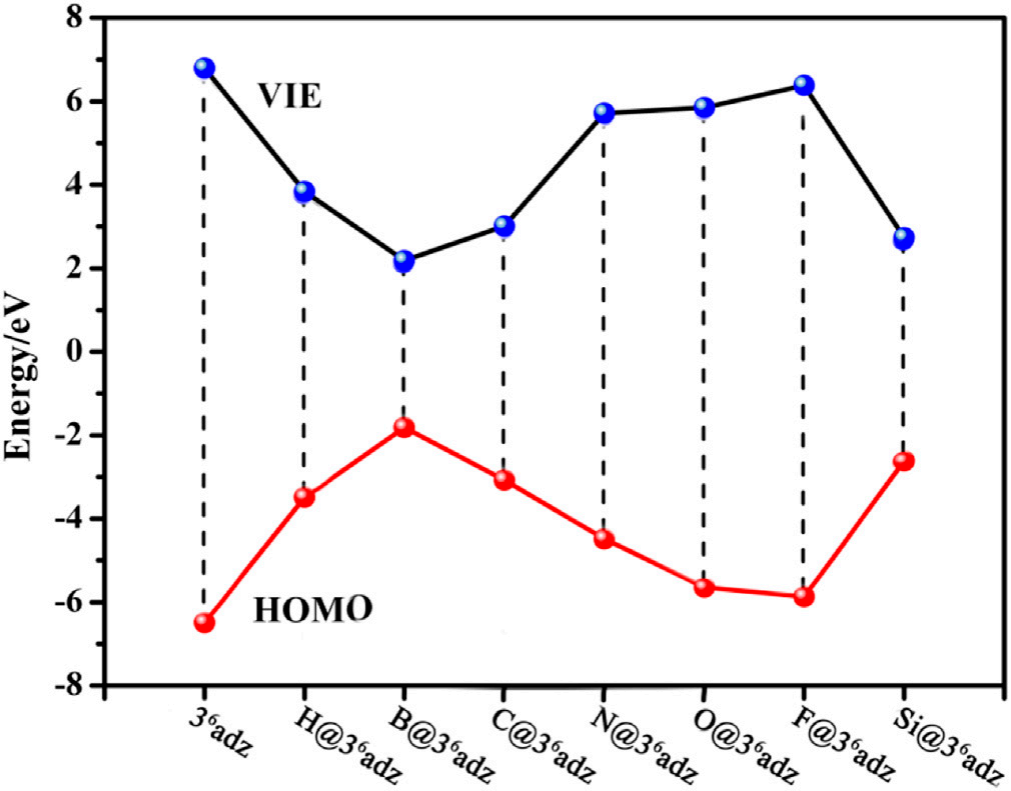
The relationship between the VIE values and HOMO levels of X@3^6^adz (X = H, B, C, N, O, F, and Si) compounds.

How to understand the low IE values of such X@3^6^adz complexes? We can find some clues from the frontier molecular orbital analysis. From Figure 3, a clear inverse correlation between the VIE values and HOMO levels of these studied compounds can be observed, that is, the higher the HOMO level is, the lower the VIE is. This is reasonable considering the fact that the valence electrons on the higher HOMOs are easier to be ionized. To be specific, all the HOMO energies (−1.81 ∼ −5.87 eV) of X@3^6^adz are much higher than that of −6.49 eV for 3^6^adz, because of the repulsion between the lone pairs of N atoms and the outmost valence electrons of X, resulting in the lower VIEs (2.18–6.38 eV) than that (6.80 eV) of 3^6^adz. In particular, B@3^6^adz exhibits the highest HOMO level of −1.81 eV, and thus possesses the lowest VIE of 2.18 eV among these X@3^6^adz complexes. This is because that the valence electron of embedded boron atom is pushed out of the cage by the lone pairs of N atoms of the complexant, forming a electride-like molecule [B^+^@3^6^adz](e^‒^) with obvious diffuse electrons in the HOMO of B@3^6^adz (see Supplementary Figure S3). Thus, the existence of diffuse excess electrons in its high-lying HOMO level results in the high reducibility of this B@3^6^adz complex.

Differently, as shown in Supplementary Figure S3, the valence electrons are accommodated into the HOMOs mainly composed of the 1*s* atomic orbital of embedded hydrogen atom in H@3^6^adz, and the *np* orbitals of C and Si atoms in X@3^6^adz (X = C and Si), which show obvious antibonding character with respect to the central atom-complexant interaction. Such antibonding HOMOs destabilize the neutral structures of X@3^6^adz (X = H, C, and Si) and result in their low VIE values (Gutsev and Boldyrev, 1987; Tkachenko et al., 2019). Hence, these 3 species also have quite low VIE values of 2.73–3.83 eV. However, it should be mentioned that the VIEs of 5.72–6.38 eV for X@3^6^adz (X = N, O, and F) are larger than that of 5.39 eV for Li atom, although their HOMOs also possess obvious antibonding character. This is attributed to the larger elertonegativities of N, O, and F atoms than H, C, and Si atoms, which hinders the ionization of the valence electrons on their *np* orbitals in the HOMOs of X@3^6^adz (X = N, O, and F).

On the other hand, the difference between the VIE and AIE values are also related to the different electron distribution in the HOMOs of X@3^6^adz. To be specific, the geometric structure of B@3^6^adz is hardly changed after its diffuse excess electron of HOMO is lost, resulting in its nearly equal VIE (2.18 eV) and AIE (2.16 eV) values. However, the destabilization of antibonding HOMOs for the neutral X@3^6^adz (X = H, C, and Si) complexes drives the embedded X atom to lose nearly one valence electron (0.667 *e* ∼ 0.867 *e*, as shown in Supplementary Table S1), forming relatively stable [X@3^6^adz]^+^ cations. After losing one electron, the formed X^+^ ion changes its interaction mode with the cage complexant, which leads to the large structural distortion and considerable difference between the VIE and AIE values of X@3^6^adz (X = H, C, and Si). Note that the AIE of H@3^6^adz is as low as 0.78 eV because the formed [H@3^6^adz]^+^ is very stable and has been identified in various synthesized ionic compounds, such as [H@3^6^adz]^+^X^‒^ (X = Cl, Br, I, and Na) (Kim et al., 1994; Springborg et al., 1996; Redko et al., 2002).

Finally, considering the diffuse excess electron in the HOMO of B@3^6^adz, it is highly expected that this superalkali also exhibits considerable nonlinear optical (NLO) response. Thus, the static electric properties of these studied X@3^6^adz compounds and 3^6^adz complexant were calculated and listed in Table 3. It is observed that B@3^6^adz has the largest dipole moment (3.326 au) and polarizability (1,599 au) among these X@3^6^adz complexes because of the existence of diffuse electrons in the HOMO of this superalkali. In particular, the first hyperpolarizability (*β*_0_) of B@3^6^adz is as large as 1.35 × 10^6^ au, which is significantly larger than those of the reported superalkalis and superalkali-based NLO materials, such as the aromatic organometallic superalkali Au_3_(Py)_3_ (3.74 × 10^4^ au) (Parida et al., 2018), superalkali-based alkalide Li_3_O^+^(calix [4]pyrrole)M^‒^ (M = Li, Na, and K) (1.18 × 10^4^–3.33 × 10^4^ au) (Sun et al., 2014a), and superalkali-based electride Li_3_O@Al_12_N_12_ (8.73 × 10^5^ au) (Sun et al., 2016b), indicating that this proposed superalkali species can indeed be considered as a new kind of NLO molecule of high performance.

**TABLE 3:** Calculated dipole moments (*µ*_0_, in au), polarizabilities (*α*_0_, in au), first hyperpolarizabilities (*β*_0_, in au), transition energies (Δ*E*, in eV), oscillator strength (*f*_0_), and the difference in the dipole moments (∆*µ*, in Debye) between the ground and crucial excited states of 3^6^adz and X@3^6^adz (X = H, B, C, N, O, F, and Si) compounds.

Species	*µ*_0_	*α*_0_	*β*_0_	Δ*E*	*f*_0_	∆*µ*
3^6^adz	0.000	240	0	5.64	0.100	1.113
H@3^6^adz	0.000	253	0	5.03	0.044	0.001
B@3^6^adz	3.326	1,599	1.35 × 10^6^	0.40	0.121	6.428
C@3^6^adz	2.074	278	4.05 × 10^3^	2.25	0.064	2.854
N@3^6^adz	1.471	257	2.84 × 10^2^	4.67	0.033	0.495
O@3^6^adz	1.558	249	6.84 × 10^2^	5.40	0.059	1.801
F@3^6^adz	1.142	255	2.43 × 10^2^	3.52	0.093	0.544
Si@3^6^adz	0.773	354	1.95 × 10^4^	1.84	0.040	5.663

To understand the eminently large *β*_0_ value of B@3^6^adz, we focus our attention on the simple two-level model (Oudar, 1977; Oudar and Chemla, 1977),

$$\beta_{0}\propto \frac{\Delta \mu \cdot f_{0}}{\Delta E^{3}}$$ (4)

where Δ*E*, *f*_0_, and Δ*µ* are the transition energy, oscillator strength, and the difference in the dipole moment between the ground state and crucial excited state, respectively. According to this two-level expression, *β*_0_ is proportional to *f*_0_ and ∆*µ*, while is inversely proportional to the cube of Δ*E*, and therefore, the transition energy is considered to be the decisive factor in the first hyperpolarizability (Sun et al., 2014a,b, 2016b,c). Hence, the Δ*E*, *f*_0_, and Δ*µ* values of the crucial excited states with the largest oscillator strength of 3^6^adz and X@3^6^adz are summarized in Table 3. It is noted that B@3^6^adz possesses extremely smaller Δ*E* and much larger *f*_0_ and ∆*µ* values than those of other X@3^6^adz (X = H, C, N, O, F, and Si) compounds, which rationalizes its largest *β*_0_ value among these studied X@3^6^adz species. In addition, the proposed C@3^6^adz and Si@3^6^adz superalkalis also show considerable *β*_0_ values of 4.05 × 10^3^ au and 1.95 × 10^4^ au, respectively, because of their relatively smaller Δ*E* values and larger Δ*µ* values.

## Conclusion

By using 3^6^adamanzane (3^6^adz) as a complexant, a series of X@3^6^adz (X = H, B, C, N, O, F, and Si) compounds were constructed and studied based on the density functional theory. It is interesting to find that the X@3^6^adz (X = H, B, C, N, and Si) complexes possess lower AIE values than the IE of Cs atoms though the X atoms and 3^6^adz possess very high IE values. Thereby, they can be regarded as a new kind of nonmetallic superalkalis. In particular, different from other complexes, the low IE of B@3^6^adz is derived from the diffuse excess electron formed by the repulsion between the valence electron of the embedded boron atom and lone pairs of N atoms of the complexant. Due to the existence of diffuse electrons, this superalkali also possesses a remarkably large *β*_0_ of 1.35 × 10^6^ au, which can serve as a new kind of NLO molecule. Hence, it is highly hoped that the theoretical design and characterization of these nonmetallic superalkali species could provide meaningful references to further design novel reducing matters or NLO materials by using such cage-like molecules as complexants.

## Data Availability

The original contributions presented in the study are included in the article/Supplementary Material, further inquiries can be directed to the corresponding author.
