# Supplementary material for: Designing Special Nonmetallic Superalkalis Based on a Cage-like Adamanzane Complexant
Source: Front Chem. 2022 Mar 14;10:853160. doi: 10.3389/fchem.2022.853160 (PMC8963935; doi:10.3389/fchem.2022.853160)
Supplement: Supplementary file 1 [file DataSheet1.PDF]

frontiers

Supplementary Material

1. Figures

Figure S1. The first four HOMOs (HOMO ~ HOMO-3) corresponding to the lone pairs of N atoms of optimized 3^6adz complexant.

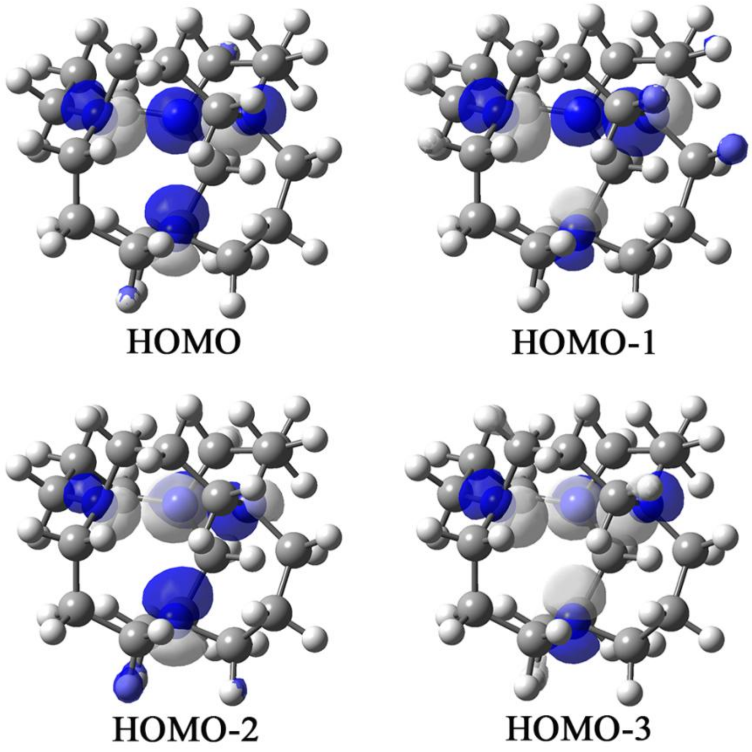

Figure S2 Optimized geometric structures of $[X@3^6adz]^+$ (X = H, B, C, N, O, F, and Si) with the symmetries shown in the parentheses.

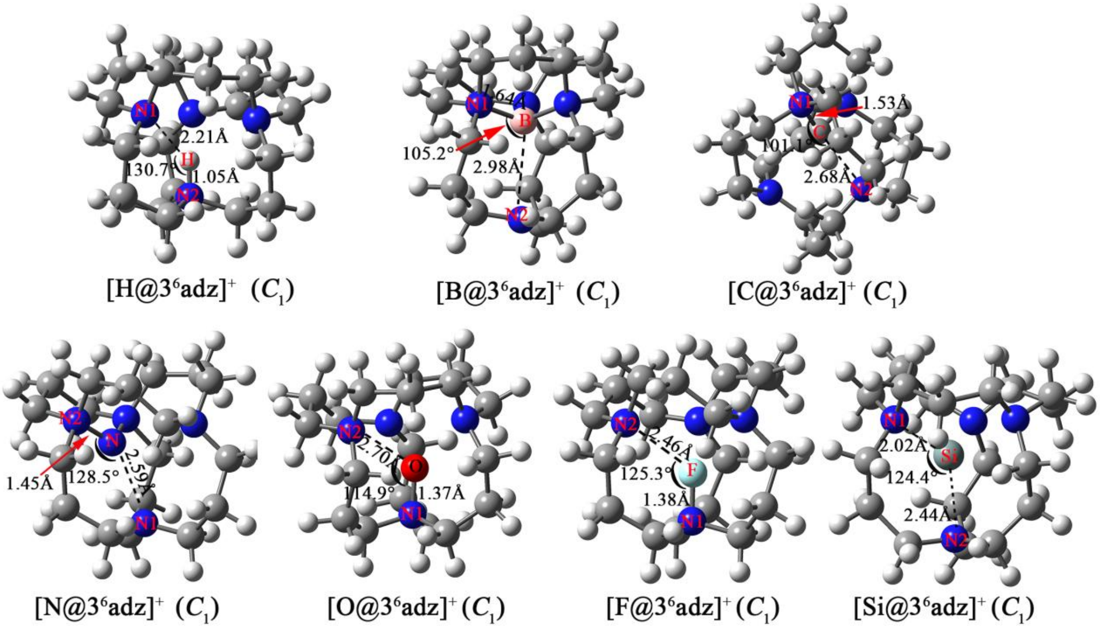

Figure S3. The HOMOs of X@3^6adz (X = H, B, C, N, O, F, and Si) with isovalue of 0.03 a.u..

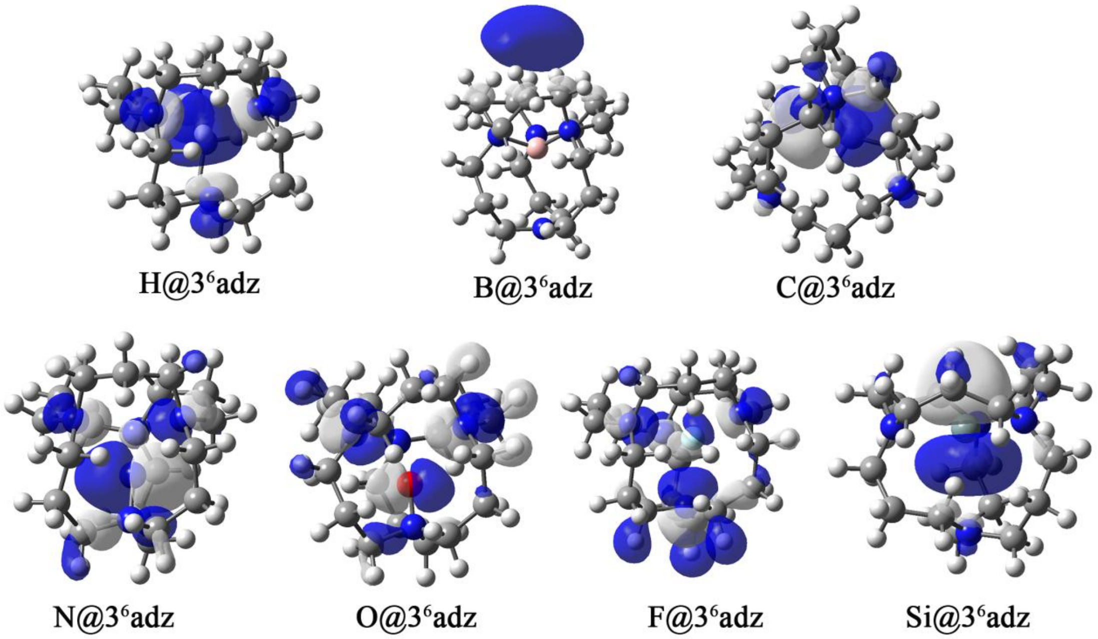

2. Tables

Table S1 The calculated vertical ionization energies (VIE, in eV), dipole moments (μ_0, in au), polarizabilities (α_0, in au), first hyperpolarizabilities (β_0, in au) of B@3^{6}adz by using different methods with the same 6-311++G(d, p) basis set.

Methods	VIE	μ_0	α_0	β_0
B3LYP	2.40	2.137	1861	1.41×10^6
PBE0	2.32	2.885	1781	1.56×10^6
*w*B97X-D	2.00	4.151	1092	3.38×10^5
M06-2X	2.19	3.648	2864	1.68×10^7
**CAM-B3LYP**	2.18	3.326	1599	1.35×10^6

Table S2 Natural charges on the central X atoms in the X@3^6adz (X = H, B, C, N, O, F, and Si) and their cations and the change in NPA charges (ΔQ, in |e|) after losing one electron.

Species	Neutral molecule	Cation	ΔQ
H@3^6adz	-0.115	0.664	0.779
B@3^6adz	0.362	0.380	0.018
C@3^6adz	-0.462	0.405	0.867
N@3^6adz	-0.331	-0.408	-0.077
O@3^6adz	-0.731	-0.472	0.259
F@3^6adz	-0.522	-0.189	0.333
Si@3^6adz	0.048	0.715	0.667

3. Cartesian Coordinates for the optimized 3^6adz, X@3^6adz, and [X@3^6adz]$^+$ (X = H, B, C, N, O, F, and Si) at the CAM-B3LYP/6-31+G(d) level

(1) 3^6adz with S_4 symmetry

N	1.22540409	1.15561809	1.06330508
C	0.64530305	2.45598419	1.35179410
H	1.40098011	3.14063024	1.79266614
H	-0.12583501	2.33150918	2.11561116
C	0.00000000	3.15594324	0.15061101
H	0.73693406	3.31892925	-0.64211005
H	-0.28217102	4.16332332	0.48476004
C	-1.27853910	2.52469719	-0.41887703
H	-2.00318015	2.43247219	0.39711303
H	-1.71752213	3.25756925	-1.13232008
N	-1.15561809	1.22540409	-1.06330508
C	-2.45598419	0.64530305	-1.35179410
H	-2.33150918	-0.12583501	-2.11561116
H	-3.14063024	1.40098011	-1.79266614
C	-3.15594324	0.00000000	-0.15061101
H	-4.16332332	-0.28217102	-0.48476004
H	-3.31892925	0.73693406	0.64211005
C	-2.52469719	-1.27853910	0.41887703
H	-3.25756925	-1.71752213	1.13232008
H	-2.43247219	-2.00318015	-0.39711303
N	-1.22540409	-1.15561809	1.06330508
C	-0.64530305	-2.45598419	1.35179410
H	-1.40098011	-3.14063024	1.79266614
H	0.12583501	-2.33150918	2.11561116
C	-0.00000000	-3.15594324	0.15061101
H	0.28217102	-4.16332332	0.48476004
H	-0.73693406	-3.31892925	-0.64211005
C	1.27853910	-2.52469719	-0.41887703
H	1.71752213	-3.25756925	-1.13232008
H	2.00318015	-2.43247219	0.39711303
N	1.15561809	-1.22540409	-1.06330508
C	2.45598419	-0.64530305	-1.35179410
H	2.33150918	0.12583501	-2.11561116
H	3.14063024	-1.40098011	-1.79266614
C	3.15594324	-0.00000000	-0.15061101
H	4.16332332	0.28217102	-0.48476004
H	3.31892925	-0.73693406	0.64211005
C	2.52469719	1.27853910	0.41887703
H	3.25756925	1.71752213	1.13232008
H	2.43247219	2.00318015	-0.39711303
C	1.29176110	0.25438002	2.19874417
H	2.05830516	0.59070105	2.93233822
H	1.63900412	-0.70513606	1.81626014

C	0.00000000	0.00000000	2.98227123
H	-0.21264302	0.84071207	3.65404628
H	0.21264302	-0.84071207	3.65404628
C	-1.29176110	-0.25438002	2.19874417
H	-2.05830516	-0.59070105	2.93233822
H	-1.63900412	0.70513606	1.81626014
C	-0.25438002	1.29176110	-2.19874417
H	0.70513606	1.63900412	-1.81626014
H	-0.59070105	2.05830516	-2.93233822
C	0.00000000	0.00000000	-2.98227123
H	0.84071207	0.21264302	-3.65404628
H	-0.84071207	-0.21264302	-3.65404628
C	0.25438002	-1.29176110	-2.19874417
H	-0.70513606	-1.63900412	-1.81626014
H	0.59070105	-2.05830516	-2.93233822

(2) H@3^6adz with S_4 symmetry

N	1.28390500	1.21285600	1.15826500
C	0.63860400	2.49033700	1.37212000
H	1.34790000	3.22800900	1.80480200
H	-0.14536800	2.35684200	2.12190000
C	0.00000000	3.14639300	0.13148500
H	0.75137700	3.28351400	-0.65249100
H	-0.26703400	4.16624400	0.43951700
C	-1.29502700	2.55447800	-0.46342800
H	-2.02203900	2.44932100	0.34859000
H	-1.71178100	3.33070400	-1.14349700
N	-1.21285600	1.28390500	-1.15826500
C	-2.49033700	0.63860400	-1.37212000
H	-2.35684200	-0.14536800	-2.12190000
H	-3.22800900	1.34790000	-1.80480200
C	-3.14639300	0.00000000	-0.13148500
H	-4.16624400	-0.26703400	-0.43951700
H	-3.28351400	0.75137700	0.65249100
C	-2.55447800	-1.29502700	0.46342800
H	-3.33070400	-1.71178100	1.14349700
H	-2.44932100	-2.02203900	-0.34859000
N	-1.28390500	-1.21285600	1.15826500
C	-0.63860400	-2.49033700	1.37212000
H	-1.34790000	-3.22800900	1.80480200
H	0.14536800	-2.35684200	2.12190000
C	0.00000000	-3.14639300	0.13148500
H	0.26703400	-4.16624400	0.43951700
H	-0.75137700	-3.28351400	-0.65249100
C	1.29502700	-2.55447800	-0.46342800
H	1.71178100	-3.33070400	-1.14349700
H	2.02203900	-2.44932100	0.34859000

N	1.21285600	-1.28390500	-1.15826500
C	2.49033700	-0.63860400	-1.37212000
H	2.35684200	0.14536800	-2.12190000
H	3.22800900	-1.34790000	-1.80480200
C	3.14639300	0.00000000	-0.13148500
H	4.16624400	0.26703400	-0.43951700
H	3.28351400	-0.75137700	0.65249100
C	2.55447800	1.29502700	0.46342800
H	3.33070400	1.71178100	1.14349700
H	2.44932100	2.02203900	-0.34859000
C	1.30563800	0.27839300	2.26405900
H	2.05946700	0.57474100	3.02628400
H	1.64630300	-0.67377900	1.85461100
C	0.00000000	0.00000000	3.02527200
H	-0.22896600	0.83634000	3.69776700
H	0.22896600	-0.83634000	3.69776700
C	-1.30563800	-0.27839300	2.26405900
H	-2.05946700	-0.57474100	3.02628400
H	-1.64630300	0.67377900	1.85461100
C	-0.27839300	1.30563800	-2.26405900
H	0.67377900	1.64630300	-1.85461100
H	-0.57474100	2.05946700	-3.02628400
C	0.00000000	0.00000000	-3.02527200
H	0.83634000	0.22896600	-3.69776700
H	-0.83634000	-0.22896600	-3.69776700
C	0.27839300	-1.30563800	-2.26405900
H	-0.67377900	-1.64630300	-1.85461100
H	0.57474100	-2.05946700	-3.02628400
H	0.00000000	0.00000000	0.00000000

(3) B@3^{6}adz with C_1 symmetry

N	-2.79921300	0.00356000	0.21869600
C	-2.61728700	0.32788700	1.60989400
H	-3.54538400	0.07464300	2.15299800
H	-2.52724900	1.41397800	1.70277300
C	-1.48970800	-0.34320500	2.43423000
H	-1.46236900	-1.40880600	2.19973800
H	-1.84019200	-0.29611500	3.47809400
C	-0.07463200	0.23075300	2.49838700
H	-0.11398300	1.31865600	2.48748900
H	0.37602700	-0.04257700	3.46501800
N	0.92288000	-0.20261700	1.44162200
C	2.21724900	0.52282000	1.72769600
H	3.01940400	0.00483000	1.19298400
H	2.43229700	0.43589200	2.79989200
C	2.21772400	1.96868800	1.28950700

H	3.22961300	2.35705600	1.46939200
H	1.54063700	2.59176200	1.88136300
C	1.95601700	2.07801700	-0.19366000
H	1.99270900	3.12419100	-0.52007800
H	2.74539300	1.53854000	-0.72548600
N	0.62525600	1.48724400	-0.61352900
C	0.61053600	1.56378500	-2.10803700
H	0.99504800	2.55024300	-2.39251000
H	-0.42450800	1.49693500	-2.43883100
C	1.40658600	0.44792600	-2.74533000
H	1.41435900	0.60256400	-3.83139300
H	2.45765100	0.44880400	-2.43700200
C	0.73166000	-0.87403800	-2.46892700
H	1.23467600	-1.69445300	-2.99257900
H	-0.29092000	-0.82455900	-2.83652200
N	0.68008200	-1.24583300	-1.01188000
C	-0.38406000	-2.33133700	-0.90959100
H	-0.31834200	-2.75876600	0.08597300
H	-0.06530700	-3.12043900	-1.61101700
C	-1.85154300	-1.98750100	-1.20520300
H	-2.29250600	-2.97113600	-1.43416100
H	-1.93775000	-1.44400800	-2.14837900
C	-2.85197500	-1.38436600	-0.17365700
H	-3.83744800	-1.58797700	-0.63336100
H	-2.82876500	-2.00019000	0.73068200
C	-2.60270200	0.94632100	-0.85325700
H	-3.58062200	1.21588300	-1.30018900
H	-2.03399900	0.44687300	-1.64017400
C	-1.90140000	2.28911700	-0.60906200
H	-2.48903800	2.90012700	0.08845000
H	-1.98290600	2.82226800	-1.56478900
C	-0.45453600	2.41237200	-0.09949000
H	-0.12656600	3.43818500	-0.33021300
H	-0.44387200	2.31847600	0.97937600
C	1.21042200	-1.64494100	1.74035700
H	0.25209700	-2.15228300	1.78779400
H	1.64536500	-1.68368800	2.74639000
C	2.13656200	-2.34074500	0.75232400
H	1.92932800	-3.41386500	0.82039000
H	3.18624400	-2.21690700	1.04986800
C	2.01891200	-1.86356300	-0.68914300
H	2.77757200	-1.09970100	-0.89834000
H	2.21311300	-2.69665900	-1.37478700
B	0.20604400	-0.01843800	-0.04709100

(4) C@3^{6}adz with C_2 symmetry

N	1.15594300	0.48087600	1.24341300

C	1.19133200	2.00466300	1.38061200
H	2.08803700	2.28069400	1.96320800
H	0.31439300	2.26294300	1.97506200
C	1.21577700	2.84434100	0.09292200
H	2.07751900	2.56404300	-0.51872300
H	1.47213900	3.85057700	0.45625700
C	0.00000000	3.10136000	-0.84633600
H	-0.80703500	3.54827900	-0.25648500
H	0.36958200	3.91369200	-1.50216300
N	-0.56113600	2.06223900	-1.67227700
C	-1.93839000	1.70281900	-1.42994400
H	-2.20715100	0.88241600	-2.09963600
H	-2.56311900	2.55374300	-1.75569000
C	-2.44157800	1.37434200	0.00311900
H	-3.50881200	1.65398000	0.00394000
H	-1.95330800	2.03977600	0.71750000
C	-2.41333700	-0.06483000	0.50435300
H	-3.26344100	-0.26455100	1.17361400
H	-2.51687800	-0.73215000	-0.34493300
N	-1.15594300	-0.48087600	1.24341300
C	-1.19133200	-2.00466300	1.38061200
H	-2.08803700	-2.28069400	1.96320800
H	-0.31439300	-2.26294300	1.97506200
C	-1.21577700	-2.84434100	0.09292200
H	-1.47213900	-3.85057700	0.45625700
H	-2.07751900	-2.56404300	-0.51872300
C	0.00000000	-3.10136000	-0.84633600
H	-0.36958200	-3.91369200	-1.50216300
H	0.80703500	-3.54827900	-0.25648500
N	0.56113600	-2.06223900	-1.67227700
C	1.93839000	-1.70281900	-1.42994400
H	2.20715100	-0.88241600	-2.09963600
H	2.56311900	-2.55374300	-1.75569000
C	2.44157800	-1.37434200	0.00311900
H	3.50881200	-1.65398000	0.00394000
H	1.95330800	-2.03977600	0.71750000
C	2.41333700	0.06483000	0.50435300
H	3.26344100	0.26455100	1.17361400
H	2.51687800	0.73215000	-0.34493300
C	1.27704600	-0.11251300	2.61501400
H	2.11872800	0.35159700	3.14267200
H	1.52486800	-1.16075900	2.45696800
C	0.00000000	0.00000000	3.44687300
H	0.04674300	0.87296100	4.10567700
H	-0.04674300	-0.87296100	4.10567700
C	-1.27704600	0.11251300	2.61501400
H	-2.11872800	-0.35159700	3.14267200

H	-1.52486800	1.16075900	2.45696800
C	0.43252900	1.24497300	-2.34578600
H	1.16517900	0.90779700	-1.61513800
H	0.95870000	1.90263600	-3.06245100
C	0.00000000	0.00000000	-3.12826800
H	0.83159300	-0.25793700	-3.79706600
H	-0.83159300	0.25793700	-3.79706600
C	-0.43252900	-1.24497300	-2.34578600
H	-1.16517900	-0.90779700	-1.61513800
H	-0.95870000	-1.90263600	-3.06245100
C	0.00000000	0.00000000	0.38663100

(5) N@3^6adz with C_1 symmetry

N	2.06893100	-0.60518800	0.07512000
C	2.08422200	-1.91187400	0.83939400
H	3.11154600	-2.11806900	1.16904500
H	1.81260100	-2.68646100	0.11883900
C	1.12521100	-1.95663900	2.03467000
H	1.35655600	-1.14154500	2.72351100
H	1.41522500	-2.87193400	2.57135400
C	-0.41118400	-2.08647600	1.83975000
H	-0.59017600	-2.86051000	1.08656600
H	-0.78638000	-2.51277100	2.79805900
N	-1.19133100	-0.91403600	1.49602000
C	-2.53274800	-1.19157400	1.04260600
H	-3.08048800	-0.24411800	1.02266200
H	-3.08515500	-1.84813800	1.75347600
C	-2.64176600	-1.83154000	-0.35413000
H	-3.69260800	-2.12623200	-0.47718400
H	-2.08512600	-2.77398600	-0.38258600
C	-2.29889400	-0.97393500	-1.58794500
H	-2.66762700	-1.53330900	-2.48035600
H	-2.90117300	-0.06153300	-1.53778000
N	-0.91639100	-0.58801300	-1.75997100
C	-0.68887500	0.52844700	-2.64800400
H	-1.16458700	0.35133800	-3.63895700
H	0.38632700	0.59318200	-2.83188200
C	-1.14607200	1.91484700	-2.15768900
H	-1.20476000	2.54970400	-3.05282000
H	-2.17355500	1.86956300	-1.78395900
C	-0.24233300	2.68937900	-1.17503000
H	-0.57943600	3.75151100	-1.20472800
H	0.76842300	2.68672200	-1.59341300
N	-0.16719000	2.22386100	0.18854900
C	1.05313200	2.54184800	0.89500600
H	0.92395900	2.26036700	1.94359900
H	1.23115400	3.63975000	0.89349000

C	2.39526000	1.91946800	0.38629700
H	3.18196700	2.55696200	0.81674300
H	2.48078400	2.05663800	-0.69527500
C	2.85043200	0.48658700	0.75500900
H	3.91181500	0.36670800	0.49637000
H	2.73431400	0.31979500	1.82704500
C	2.49623900	-0.81632300	-1.35209400
H	3.46884300	-1.33124300	-1.39147400
H	2.63006000	0.19232500	-1.74052900
C	1.47485500	-1.58275800	-2.23123200
H	1.81323100	-2.62388000	-2.30923100
H	1.59471600	-1.18124800	-3.24378200
C	-0.03955000	-1.72923300	-1.92101300
H	-0.40555400	-2.37640100	-2.75125900
H	-0.13156600	-2.33573700	-1.01764700
C	-0.99678000	0.14704800	2.45686000
H	0.08053900	0.29362200	2.50875000
H	-1.33251700	-0.16804700	3.47204000
C	-1.60079200	1.53946700	2.22270900
H	-1.18881500	2.15374800	3.03421200
H	-2.68244400	1.52597400	2.41054600
C	-1.42926000	2.27986000	0.88323900
H	-2.17411900	1.87156600	0.19693200
H	-1.74899000	3.33281200	1.07216700
N	0.78690000	-0.02758400	0.09271800

(6) O@3^{6}adz with C_1 symmetry

N	-2.12846500	0.16018100	-0.27015600
C	-2.52191000	1.60591400	-0.09006400
H	-3.60333200	1.67595000	0.08888300
H	-2.30561200	2.10011100	-1.03949300
C	-1.74495600	2.29244800	1.04310300
H	-1.93303300	1.77273800	1.98610400
H	-2.24560600	3.26477200	1.15736700
C	-0.23500700	2.64055700	0.90310000
H	-0.08128000	3.06059900	-0.09566900
H	-0.07195300	3.48630400	1.60967500
N	0.77603600	1.62549000	1.13344100
C	2.10420000	1.99133100	0.70731500
H	2.80339200	1.26267700	1.12857500
H	2.41452500	2.97964900	1.11794000
C	2.33434700	2.04036700	-0.81585900
H	3.33696100	2.46717000	-0.95711700
H	1.65813100	2.76741400	-1.27705400
C	2.33035600	0.72327200	-1.61792900
H	2.75454400	0.96320600	-2.62185100

H	3.04732400	0.04268400	-1.14812700
N	1.07018600	0.02367600	-1.75477800
C	1.18520600	-1.34122700	-2.21232000
H	1.79204500	-1.40694400	-3.14415700
H	0.18359600	-1.69121300	-2.48039200
C	1.76608800	-2.34845400	-1.20530900
H	2.01929000	-3.25058300	-1.77931700
H	2.72313100	-1.98979300	-0.81408500
C	0.85641600	-2.82862400	-0.05824200
H	1.35047600	-3.72140600	0.39290300
H	-0.06815300	-3.20025900	-0.51155500
N	0.50773400	-1.87006700	0.96135700
C	-0.71113200	-2.15213000	1.68434500
H	-0.77757900	-1.46346400	2.52959500
H	-0.67530900	-3.17217500	2.12746000
C	-2.05818000	-2.08912800	0.90481900
H	-2.75933500	-2.71214700	1.47853900
H	-1.95243000	-2.60353600	-0.05422500
C	-2.81671900	-0.76454700	0.70117300
H	-3.84390600	-0.96156800	0.36332800
H	-2.86739300	-0.21633600	1.64459200
C	-2.31513400	-0.30392500	-1.68711400
H	-3.35500300	-0.13356400	-1.99822500
H	-2.14907100	-1.37838900	-1.64042200
C	-1.35516700	0.31721200	-2.72484400
H	-1.84949100	1.18707100	-3.17655100
H	-1.30360200	-0.41672600	-3.53825500
C	0.06859100	0.83990700	-2.40774100
H	0.44475800	1.18690300	-3.39901700
H	-0.05219300	1.73458500	-1.79982400
C	0.63050600	0.97928900	2.41380300
H	-0.41115500	0.66774100	2.47351100
H	0.79452300	1.69268600	3.25554700
C	1.47799400	-0.26320600	2.72785200
H	1.05410400	-0.67400900	3.65371700
H	2.49821100	0.03487700	3.00221300
C	1.65321500	-1.38550900	1.69243600
H	2.36597000	-1.02482800	0.94718800
H	2.17474700	-2.21498400	2.22761600
O	-0.82262700	0.07356600	0.00630000

(7) F@3^6adz with C_1 symmetry

N	-0.06095203	1.77756211	-1.26667784
C	1.18937851	1.62868934	-1.96750594
H	1.39013401	2.51612340	-2.60705536
H	1.09757711	0.78247091	-2.65149975
C	2.49579648	1.43023273	-1.14037679

H	2.53292241	2.15803115	-0.32437705
H	3.30470214	1.74497496	-1.81476820
C	2.97479570	0.04278753	-0.63767802
H	2.88915521	-0.67609822	-1.45553492
H	4.05106057	0.14097998	-0.41097951
N	2.31864764	-0.55072769	0.52389976
C	2.16085801	-2.00037196	0.59720724
H	1.76828281	-2.23475491	1.58824142
H	3.16209952	-2.45892672	0.54380035
C	1.28953985	-2.70577403	-0.47743487
H	1.61084961	-3.75588317	-0.41573151
H	1.60914130	-2.38039446	-1.47036231
C	-0.26633419	-2.78807950	-0.41367082
H	-0.51651793	-3.71395369	-0.97850841
H	-0.55237164	-2.99841132	0.62206043
N	-1.09070150	-1.69657875	-0.89754434
C	-2.46681015	-1.72249876	-0.46974253
H	-2.93850352	-2.71365961	-0.65685151
H	-3.02220268	-1.01156873	-1.08920179
C	-2.72937836	-1.37295363	1.00880092
H	-3.80056493	-1.55356568	1.17243162
H	-2.22648185	-2.09482147	1.66033177
C	-2.47669455	0.06322652	1.51102959
H	-2.97771625	0.13684089	2.50468954
H	-3.01723214	0.75369961	0.85534552
N	-1.10262801	0.51938517	1.61730140
C	-0.97856625	1.93803341	1.87825934
H	0.04242779	2.13682711	2.21610962
H	-1.64044689	2.25112131	2.71715982
C	-1.26052292	2.87729983	0.69128970
H	-1.31190491	3.89193897	1.10928109
H	-2.26051682	2.69180430	0.28692344
C	-0.22701521	2.95270564	-0.45354324
H	-0.51523101	3.83355660	-1.07532922
H	0.74273856	3.20741374	-0.01072152
C	-1.29431224	1.30353367	-1.85108008
H	-1.77061441	2.10988360	-2.45449425
H	-1.98002250	1.10630325	-1.02506740
C	-1.32720597	0.05840594	-2.75076858
H	-0.80437524	0.25683807	-3.69556861
H	-2.38297866	-0.03247671	-3.03771923
C	-0.81642162	-1.31150636	-2.26141691
H	-1.19584797	-2.06715883	-2.98686619
H	0.26685165	-1.30913064	-2.36885129
C	2.19512171	0.29443929	1.70218387
H	1.96721984	1.29113218	1.33024280
H	3.17555131	0.35624153	2.20697406

C	1.15611573	-0.05389867	2.77847466
H	1.20978702	0.78305020	3.48616290
H	1.50396566	-0.91918959	3.35729559
C	-0.31493149	-0.36805936	2.44946905
H	-0.33270577	-1.34374201	1.96771010
H	-0.80093846	-0.50396876	3.44291843
F	0.60574067	-0.25847589	-0.17089142

(8) Si@3^6adz with C_1 symmetry

N	-1.75787400	-0.75378100	-1.18057300
C	-1.89016800	-2.20171500	-0.87589500
H	-2.38138000	-2.68649500	-1.74070300
H	-2.54482400	-2.30054000	-0.01176400
C	-0.57700900	-3.01207800	-0.62204700
H	0.08916200	-2.87737000	-1.48184600
H	-0.91126100	-4.05502500	-0.70216800
C	0.27690500	-2.99630700	0.70240000
H	-0.40131900	-3.00014700	1.55615800
H	0.82328300	-3.96137600	0.69238600
N	1.23809900	-1.91162600	0.89233700
C	1.41587900	-1.32490300	2.21681500
H	2.36719100	-0.78705000	2.20360400
H	1.52739900	-2.12242800	2.97002000
C	0.32722500	-0.36331600	2.88487600
H	0.81546300	-0.18386600	3.86649700
H	-0.57444800	-0.93723400	3.08792800
C	-0.07394400	1.12134200	2.51134100
H	-0.59546100	1.50241800	3.39943000
H	0.84402500	1.70204700	2.41854600
N	-0.92409000	1.43880500	1.31566300
C	-0.60610000	2.75485700	0.68789200
H	-0.91617900	3.55113200	1.38678300
H	-1.26898300	2.83698800	-0.18003000
C	0.83184700	3.08935200	0.24484500
H	0.83624300	4.18762300	0.20089100
H	1.54110900	2.85500000	1.04247000
C	1.45857200	2.67765000	-1.12554300
H	2.35482200	3.32326500	-1.19078600
H	0.79881800	3.01057000	-1.93265900
N	1.85603000	1.31465200	-1.37446500
C	1.20604300	0.55755200	-2.43386600
H	1.41974000	-0.49698400	-2.26636500
H	1.69287500	0.81865500	-3.39291400
C	-0.33181000	0.74284700	-2.67087900
H	-0.45981600	1.11057900	-3.70125400
H	-0.71789300	1.53126100	-2.02242200
C	-1.25353100	-0.48609700	-2.55203500

H	-2.11047300	-0.37042700	-3.23679800
H	-0.71708800	-1.37943300	-2.88323400
C	-2.94173000	0.06921400	-0.83785900
H	-3.84049900	-0.39598900	-1.27183900
H	-2.81090700	1.03054700	-1.34477600
C	-3.21421800	0.30229300	0.66518300
H	-3.21872700	-0.66004900	1.17440000
H	-4.24687600	0.69236500	0.70251800
C	-2.39257800	1.31934700	1.51998900
H	-2.83327100	2.31778600	1.38214900
H	-2.57117200	1.04405000	2.56393900
C	2.33385300	-1.92639600	-0.06960800
H	1.88441300	-2.18602200	-1.02973400
H	3.04630200	-2.74486000	0.15417400
C	3.19451500	-0.65856200	-0.28098800
H	3.75345800	-0.82638400	-1.21102500
H	3.95854000	-0.63859600	0.50668400
C	2.60501100	0.75863100	-0.26315500
H	2.00843800	0.82719600	0.65481600
H	3.47608900	1.42631900	-0.12449200
Si	-0.37246500	-0.30829600	0.28442100

(9) [H@3^{6}adz]$^+$ with C_1 symmetry

N	1.77181200	-0.22800900	-0.87476600
C	1.61840100	-1.24648200	-1.92385400
H	2.60831200	-1.51317300	-2.33474600
H	1.05796200	-0.81155500	-2.75294600
C	0.90354900	-2.52436700	-1.48270400
H	1.40269100	-2.97161400	-0.61669400
H	1.04055200	-3.25526700	-2.28904000
C	-0.60766300	-2.42220500	-1.25826500
H	-1.04744900	-1.95148100	-2.14110000
H	-1.02088900	-3.44775000	-1.22558700
N	-1.06683800	-1.67847400	-0.07568800
C	-2.50740700	-1.41452200	-0.18230900
H	-2.89218700	-1.17087900	0.81256900
H	-3.03966900	-2.33336300	-0.48955400
C	-2.89640200	-0.28137200	-1.13197600
H	-3.99230100	-0.27522700	-1.17624800
H	-2.58026700	-0.50311200	-2.15474300
C	-2.48435100	1.13854400	-0.73113700
H	-3.02524500	1.84197500	-1.39120600
H	-2.85835800	1.33324500	0.27995300
N	-1.04811400	1.46541400	-0.75943500
C	-0.83431000	2.79181700	-0.18049500
H	-1.61481000	3.50621200	-0.49598400

H	0.11362400	3.18949500	-0.55181400
C	-0.76607500	2.74829700	1.34471800
H	-0.61142100	3.76931500	1.71315700
H	-1.71599400	2.42720200	1.78121800
C	0.40141800	1.93322900	1.89306900
H	0.49060500	2.07924100	2.97391700
H	1.32773700	2.27977900	1.43603200
N	0.35179800	0.43538400	1.69144500
C	1.62634900	-0.18335400	2.20029300
H	1.46596700	-1.26029900	2.23400500
H	1.74945800	0.16903000	3.22928800
C	2.86854800	0.10915700	1.36677100
H	3.71934900	-0.26812700	1.94547400
H	3.04725200	1.18331300	1.26913300
C	2.88320200	-0.59222200	0.00948300
H	3.85409400	-0.40312500	-0.48320000
H	2.83581000	-1.67029200	0.18822200
C	1.99470700	1.11305600	-1.42515000
H	2.96889500	1.15942500	-1.94633000
H	2.08397700	1.79724200	-0.57680300
C	0.93140100	1.66460400	-2.38537700
H	1.13489000	1.31468300	-3.40338300
H	1.09078100	2.74768200	-2.43388100
C	-0.54656600	1.34933300	-2.13065900
H	-1.13883800	1.98690100	-2.81318800
H	-0.73099600	0.32190100	-2.43932300
C	-0.75276300	-2.42261800	1.13876600
H	0.30229800	-2.70017100	1.08521600
H	-1.31130000	-3.37611200	1.19079900
C	-0.99477400	-1.64705500	2.44421400
H	-0.35045400	-2.06830500	3.22258800
H	-2.01523800	-1.81670800	2.80424600
C	-0.85502700	-0.12584800	2.39008800
H	-1.70690400	0.30318000	1.87005700
H	-0.84375600	0.27497000	3.40865000
H	0.24779700	0.22360300	0.66750500

(10) [B@3^{6}adz]$^+$ with C_1 symmetry

N	2.72475100	0.02218100	0.18145600
C	2.65245100	-0.44064300	1.53794900
H	3.59257300	-0.20017600	2.06424300
H	2.60788800	-1.53387300	1.53212300
C	1.53652800	0.10324000	2.46599800
H	1.50586600	1.19249000	2.39143800
H	1.89716400	-0.09079100	3.48687600
C	0.12450800	-0.48175600	2.46670400
H	0.17268000	-1.56103500	2.33896200

H	-0.32542100	-0.31471000	3.45646900
N	-0.89029000	0.05174900	1.45941400
C	-2.17999100	-0.70202400	1.70663400
H	-2.98134000	-0.13384100	1.23039300
H	-2.37661900	-0.72021400	2.78360900
C	-2.17009500	-2.10833600	1.14176900
H	-3.15269600	-2.55422900	1.32674000
H	-1.45255700	-2.75123600	1.65866300
C	-1.93213700	-2.09519900	-0.35422000
H	-1.94558600	-3.11389300	-0.75381500
H	-2.73087200	-1.53252200	-0.84057200
N	-0.62041800	-1.42981800	-0.73959700
C	-0.62632000	-1.36782300	-2.23520400
H	-0.98317900	-2.33268000	-2.60894100
H	0.40235000	-1.23766600	-2.56609400
C	-1.45570400	-0.21325700	-2.75591600
H	-1.48045600	-0.26832800	-3.84933300
H	-2.49779300	-0.26467800	-2.42791400
C	-0.79668200	1.09222800	-2.37069900
H	-1.30972200	1.95038900	-2.81366000
H	0.22108700	1.08453800	-2.75253500
N	-0.72185100	1.33221900	-0.88641700
C	0.33687400	2.42664600	-0.71972000
H	0.27960400	2.78122000	0.30488800
H	0.00050900	3.25883500	-1.35869700
C	1.80101200	2.11876900	-1.05876800
H	2.23218300	3.11957900	-1.21147700
H	1.88079800	1.65305900	-2.04330000
C	2.79928900	1.43590400	-0.08029600
H	3.78818600	1.68297700	-0.50835500
H	2.76402200	1.96752600	0.87556600
C	2.62415000	-0.83789400	-0.96410600
H	3.62578500	-1.07201100	-1.37379700
H	2.10739700	-0.28028600	-1.74937500
C	1.92005500	-2.19961400	-0.86003300
H	2.51601700	-2.87938500	-0.23798600
H	1.99052100	-2.63175600	-1.86561500
C	0.48741100	-2.38674800	-0.33428900
H	0.16686100	-3.39226900	-0.64664600
H	0.50303800	-2.38665600	0.74837300
C	-1.16041300	1.46071100	1.89185000
H	-0.19823800	1.96023900	1.92628200
H	-1.53892600	1.42392700	2.91802400
C	-2.13383400	2.24221000	1.01277800
H	-1.92930200	3.30291800	1.18515900
H	-3.16551200	2.08771400	1.34124700
C	-2.05277200	1.92599100	-0.48156400

H	-2.80444500	1.18763300	-0.76393700
H	-2.24873200	2.83312800	-1.06397400
B	-0.24489400	0.01946300	-0.05804500

(11) [C@3^{6}adz]$^+$ with C_1 symmetry

N	-1.19066200	-0.30336100	1.23132500
C	-2.15394200	0.88597900	1.29257400
H	-2.79778800	0.68518500	2.15503400
H	-2.77539700	0.82673100	0.39867700
C	-1.59437700	2.30466300	1.43749100
H	-0.92376200	2.37897400	2.29593000
H	-2.46387100	2.91165600	1.72080000
C	-0.97833400	2.97694200	0.20156500
H	-1.76453300	3.08250400	-0.55271300
H	-0.70606600	4.00829100	0.49386900
N	0.13949500	2.28545000	-0.39591000
C	0.12000700	2.25076900	-1.84060400
H	1.10801500	1.97037100	-2.21043200
H	-0.09019200	3.24690700	-2.26955600
C	-0.92666100	1.27456600	-2.41808300
H	-1.05684800	1.53354600	-3.47630300
H	-1.90395000	1.46190200	-1.96056700
C	-0.59105200	-0.22016700	-2.41944900
H	-1.13707100	-0.72362300	-3.22416400
H	0.46804700	-0.34144600	-2.62073000
N	-0.89541300	-1.03792400	-1.16962400
C	-0.16788900	-2.37493100	-1.29500200
H	-0.57925600	-2.84840200	-2.19477500
H	-0.48130600	-2.97027700	-0.43590200
C	1.36526800	-2.38622600	-1.40871000
H	1.57654100	-3.41859800	-1.71793900
H	1.68653500	-1.78841800	-2.26478500
C	2.33660200	-2.14465500	-0.21580500
H	3.31288600	-2.47061900	-0.61893800
H	2.09594700	-2.85835000	0.57853500
N	2.46344800	-0.83481500	0.36590900
C	2.28602600	-0.69513900	1.78484700
H	2.46151300	0.34888400	2.05640700
H	3.07124800	-1.26619000	2.30801900
C	0.95485800	-1.16237900	2.43407500
H	1.18112400	-1.30851200	3.49865900
H	0.68948400	-2.15637400	2.06340900
C	-0.24360900	-0.21278300	2.41719000
H	-0.87073600	-0.35313600	3.30463600
H	0.12989900	0.80450900	2.43170600
C	-1.98827600	-1.56735400	1.38004700
H	-2.57869400	-1.49966500	2.29735200
H	-1.25815100	-2.36251700	1.52033300

C	-2.88432200	-1.86500700	0.17802700
H	-3.89252600	-1.47695400	0.34606000
H	-2.98873000	-2.95072100	0.10472600
C	-2.37676500	-1.28376300	-1.14387800
H	-2.62900500	-1.94253900	-1.97856400
H	-2.84529400	-0.32125200	-1.34462900
C	1.38702700	2.44427000	0.33722800
H	1.18174900	2.14102700	1.36824400
H	1.66542100	3.51404000	0.40014500
C	2.65376600	1.70977800	-0.14558100
H	3.40891000	1.86746200	0.63426900
H	3.04599800	2.25024400	-1.01528800
C	2.64111000	0.23934000	-0.58493900
H	1.86349600	0.15843000	-1.34887500
H	3.60072300	0.08043500	-1.10843700
C	-0.24317500	-0.33706200	0.03298600

(12) [N@3^{6}adz]$^+$ with C_1 symmetry

N	-1.51618300	0.26373600	-1.13906400
C	-0.79205600	1.05931700	-2.22719400
H	-1.57886400	1.36355400	-2.92353000
H	-0.14731500	0.36037100	-2.75000900
C	0.02794400	2.27339100	-1.78686900
H	-0.54553100	2.88481300	-1.08678900
H	0.11094400	2.88006100	-2.69837200
C	1.48574500	2.07874000	-1.29777200
H	1.98494400	1.38754800	-1.98413700
H	1.98614900	3.05579700	-1.44913700
N	1.68328600	1.60536000	0.05744600
C	2.97919000	1.02320800	0.35173500
H	3.04502700	0.90742100	1.44065300
H	3.80373900	1.71199100	0.07791300
C	3.29364700	-0.33950900	-0.29276900
H	4.35539700	-0.52101100	-0.08464200
H	3.24391500	-0.26418100	-1.38370800
C	2.57300200	-1.61405800	0.19100600
H	3.17990600	-2.47136000	-0.16376500
H	2.62734600	-1.64564700	1.28680100
N	1.17536900	-1.81134100	-0.18665100
C	0.54020400	-2.91431600	0.50556300
H	1.26648100	-3.69823700	0.77954300
H	-0.17719800	-3.40344400	-0.15965700
C	-0.19803900	-2.48112700	1.77333600
H	-0.64562600	-3.37787600	2.22270000
H	0.51127000	-2.11618300	2.51997700
C	-1.37461100	-1.51115900	1.61784500
H	-1.89541500	-1.47613300	2.57849400

H	-2.08496000	-1.89927300	0.89360000
N	-1.12774400	-0.02244400	1.27212100
C	-2.38463700	0.73726500	1.58096900
H	-2.10361900	1.78939000	1.61532000
H	-2.76648500	0.43936800	2.56051000
C	-3.41117500	0.50946000	0.48043100
H	-4.28857400	1.12965100	0.68700400
H	-3.76863800	-0.52481000	0.47179000
C	-2.84782900	0.91934600	-0.87217400
H	-3.53473500	0.67967600	-1.68788300
H	-2.65482400	1.99362500	-0.88010800
C	-1.71138300	-1.14324500	-1.67363100
H	-2.41155300	-1.06118600	-2.51134600
H	-2.22911200	-1.69390900	-0.89455900
C	-0.47332200	-1.92505500	-2.16942100
H	-0.36938000	-1.75005000	-3.24584000
H	-0.76861800	-2.97748000	-2.10171000
C	0.95609700	-1.74856500	-1.61447000
H	1.55898700	-2.50794000	-2.15339000
H	1.33194600	-0.78207300	-1.94747600
C	1.16304300	2.51003300	1.05896600
H	0.44103600	3.16609800	0.57457900
H	1.95107600	3.18028600	1.44724400
C	0.45959800	1.85301700	2.27390100
H	-0.35421900	2.50330200	2.61324400
H	1.15763900	1.82796600	3.12047500
C	0.02446300	0.39822900	2.17122300
H	0.85605100	-0.18263400	1.78681900
H	-0.24502000	0.03905600	3.16911600
N	-0.60065100	0.38520600	-0.02724900

(13) $[O@3^6adz]^+$ with C_1 symmetry

N	-1.44817900	-0.87059600	1.07946500
C	-0.78813500	-2.05991800	1.75650100
H	-1.59508300	-2.64688400	2.20656700
H	-0.17557900	-1.65715800	2.56089600
C	0.06562800	-2.93859500	0.83546900
H	-0.52822200	-3.28058600	-0.01626800
H	0.24488800	-3.84472700	1.42796700
C	1.47063800	-2.46804600	0.38135600
H	2.01607700	-2.12316700	1.26601900
H	1.99527200	-3.38569400	0.04823600
N	1.55801700	-1.43588600	-0.63691700
C	2.85889600	-0.79491800	-0.74433100
H	2.89441400	-0.26723700	-1.70284600
H	3.67098800	-1.54638600	-0.79197700
C	3.22007000	0.20682500	0.36786700
H	4.27243900	0.46832200	0.20104200

H	3.21711000	-0.29259200	1.34169800
C	2.47999700	1.55745000	0.45656200
H	3.07320700	2.19783800	1.14057900
H	2.53206500	2.04676600	-0.52355500
N	1.08009600	1.54721100	0.88039000
C	0.42984700	2.83751300	0.73268200
H	1.11430300	3.67045300	0.97825300
H	-0.38787900	2.90665800	1.45550700
C	-0.15940300	3.10654200	-0.65970900
H	-0.54652900	4.13284600	-0.64710500
H	0.62632000	3.11239600	-1.41958400
C	-1.34606300	2.24128300	-1.11026900
H	-1.79334800	2.72232600	-1.99604700
H	-2.11671300	2.25272800	-0.33634900
N	-1.09256000	0.84214400	-1.48494900
C	-2.27695600	0.06548800	-1.83290100
H	-1.94267700	-0.86832100	-2.28555600
H	-2.85505100	0.61166400	-2.59376700
C	-3.20843500	-0.25023300	-0.65053200
H	-4.11998400	-0.68249600	-1.07879300
H	-3.53787400	0.66800400	-0.15639800
C	-2.71406700	-1.27480800	0.37155300
H	-3.48251700	-1.46159500	1.12748400
H	-2.48128100	-2.21745900	-0.12691200
C	-1.69134400	0.25602500	2.04740300
H	-2.44755700	-0.08199800	2.76375100
H	-2.13220100	1.04444000	1.44228100
C	-0.47406800	0.79621000	2.81342300
H	-0.32430300	0.19679300	3.71828400
H	-0.79966100	1.77193800	3.19075600
C	0.92406800	0.91070400	2.17548400
H	1.54772300	1.40829300	2.94570200
H	1.32168600	-0.09854900	2.07510300
C	0.93818000	-1.82005600	-1.88830300
H	0.02343600	-2.35821200	-1.64242700
H	1.56509500	-2.53049800	-2.46205500
C	0.56115000	-0.66990900	-2.84173000
H	-0.18076300	-1.04766600	-3.55460300
H	1.42862900	-0.41659600	-3.46233600
C	0.14753700	0.67680700	-2.25321300
H	0.93441700	1.00841700	-1.58241600
H	0.09700900	1.39313700	-3.08898900
O	-0.55433700	-0.45693800	0.12100900

(14) [F@3^{6}adz]$^+$ with C_1 symmetry

N	0.40061200	-1.55660500	-1.32724500
C	-0.83253900	-1.82286700	-2.04022700

H	-0.77038700	-2.75665200	-2.63183100
H	-0.99990500	-1.02128800	-2.76467800
C	-2.10747200	-1.96123500	-1.17012200
H	-1.96662900	-2.72066700	-0.39639200
H	-2.88226800	-2.37850300	-1.82529100
C	-2.78219600	-0.71106700	-0.58429400
H	-2.91353900	0.05005700	-1.35392000
H	-3.76518600	-0.96165500	-0.17512000
N	-2.04906000	-0.02185400	0.54283000
C	-2.46062400	1.41298300	0.76004900
H	-2.08340100	1.68935600	1.74464300
H	-3.55384600	1.40975800	0.79311500
C	-1.94621300	2.38400700	-0.31520100
H	-2.50008100	3.30783500	-0.11028400
H	-2.29231900	2.07255500	-1.30442700
C	-0.45114000	2.80078100	-0.35594000
H	-0.43318300	3.73184900	-0.95590600
H	-0.15572700	3.09405900	0.65595300
N	0.53014600	1.85352900	-0.86248100
C	1.89995800	2.20692600	-0.51388300
H	2.10977100	3.26792600	-0.75077300
H	2.56797900	1.62151200	-1.15032400
C	2.29449300	1.95947100	0.95003200
H	3.28364100	2.41613100	1.07820300
H	1.64618500	2.52945600	1.62377800
C	2.44803600	0.50731200	1.43656500
H	3.00406300	0.54065200	2.39537300
H	3.09977900	-0.01585000	0.73188400
N	1.23522200	-0.29600100	1.59929600
C	1.52764200	-1.71468200	1.76628400
H	0.62914500	-2.20913000	2.15256500
H	2.30784700	-1.86931500	2.53720900
C	1.95936500	-2.45866200	0.49388900
H	2.29424500	-3.45163700	0.81855900
H	2.85043900	-1.99582800	0.06113300
C	0.89900200	-2.71262200	-0.59059200
H	1.31841000	-3.45985000	-1.29409100
H	0.04629700	-3.20614000	-0.11016300
C	1.45724200	-0.89485400	-2.08065800
H	1.93244100	-1.59618600	-2.79571600
H	2.22997000	-0.63799500	-1.35546900
C	1.13072600	0.37837800	-2.87765200
H	0.62471000	0.11799900	-3.81525100
H	2.10402800	0.76878700	-3.19615700
C	0.29732500	1.51364100	-2.25696800
H	0.41624200	2.39876200	-2.91394000
H	-0.75497700	1.22817800	-2.34008500
C	-1.94506800	-0.86484800	1.78331900

H	-1.61420300	-1.83343400	1.41605600
H	-2.96558300	-0.96819100	2.16390800
C	-1.01038100	-0.37089800	2.89842400
H	-0.88620100	-1.25737300	3.52999700
H	-1.54533500	0.34537800	3.53135800
C	0.36959000	0.27052200	2.61616500
H	0.19451700	1.31151800	2.33700800
H	0.86824000	0.30940600	3.60570600
F	-0.75001000	0.08237100	0.09793700

(15) [Si@3^{6}adz]$^+$ with C_1 symmetry

N	-0.04721300	2.09995000	-0.47921100
C	0.00517300	2.94674500	0.74154100
H	-0.35990000	3.95206300	0.49117900
H	1.05345300	3.04339000	1.03370400
C	-0.80489000	2.38918300	1.92219100
H	-1.84112700	2.19907800	1.61930500
H	-0.86599600	3.19302300	2.66402900
C	-0.22571400	1.16854500	2.65454600
H	0.83853900	1.33187600	2.83531100
H	-0.71018900	1.07683100	3.63612800
N	-0.37125700	-0.13694600	1.94517600
C	0.69111900	-1.10382000	2.41528900
H	0.35499500	-2.10860300	2.15488800
H	0.69087200	-1.05042300	3.51121200
C	2.15295000	-0.91743400	1.92205400
H	2.74904400	-1.42862800	2.69232400
H	2.44678100	0.13377700	1.98110600
C	2.69849900	-1.55609600	0.59331800
H	3.79093200	-1.44032800	0.66431600
H	2.50224600	-2.63343400	0.64303700
N	2.26076300	-1.09458700	-0.71530000
C	1.60749800	-2.07978900	-1.58187300
H	2.23224200	-2.98487900	-1.61225300
H	1.60362200	-1.67996000	-2.59985200
C	0.15935700	-2.56439800	-1.24104600
H	0.12138200	-3.58520700	-1.64179200
H	0.06081000	-2.69259500	-0.16054100
C	-1.08691000	-1.84003700	-1.84406200
H	-1.75201300	-2.61214600	-2.26461600
H	-0.74341800	-1.24674800	-2.69530700
N	-1.91038200	-0.95890200	-0.99469600
C	-2.58134900	0.08214000	-1.77470800
H	-3.38970400	0.49528500	-1.16346900
H	-3.06295100	-0.35474100	-2.66674200
C	-1.71295900	1.25901100	-2.26834300
H	-2.32589400	1.77503500	-3.01696700

H	-0.84333800	0.88817400	-2.81981100
C	-1.29603900	2.34877200	-1.26427000
H	-1.16686400	3.30030700	-1.79788800
H	-2.10320800	2.50083200	-0.54227300
C	1.17946400	2.26877000	-1.34886300
H	1.37245000	3.34656700	-1.40509800
H	0.90613100	1.94235800	-2.35439400
C	2.50388100	1.55932300	-0.92434200
H	2.71030200	1.74240900	0.13333900
H	3.26836600	2.13340000	-1.46945800
C	2.89757300	0.07341700	-1.31160600
H	2.79493600	-0.01016700	-2.39796100
H	3.97666600	0.03082100	-1.10108100
C	-1.73488600	-0.70416600	2.18886700
H	-2.44981400	0.10811600	2.02933100
H	-1.79053200	-0.97107900	3.25367500
C	-2.15395800	-1.91921300	1.34974300
H	-2.93389700	-2.43014600	1.92734800
H	-1.34136400	-2.64504500	1.27917100
C	-2.76466700	-1.65166300	-0.03728200
H	-3.10505200	-2.61945600	-0.44224900
H	-3.66846300	-1.05182500	0.11232100
Si	0.30369500	0.17355100	0.00366600
